# Evolutionary and Experimental Loss of Gene Body Methylation and Its Consequence to Gene Expression

**DOI:** 10.1534/g3.119.400365

**Published:** 2019-05-30

**Authors:** Adam J. Bewick, Yinwen Zhang, Jered M. Wendte, Xiaoyu Zhang, Robert J. Schmitz

**Affiliations:** * Department of Plant Biology,; † Institute of Bioinformatics, and; ‡ Department of Genetics, University of Georgia, Athens, GA 30602

**Keywords:** DNA methylation, gene body methylation, gene expression, epigenetics

## Abstract

In flowering plants, gene body methylation (gbM) is associated with a subset of constitutively expressed genes. It has been proposed that gbM modulates gene expression. Here, we show that there are no consistent and direct differences to expression following the loss of gbM. By comparing expression of gbM genes in *Arabidopsis thaliana* accessions to orthologous genes in two *Eutrema salsugineum* genotypes, we identified both positive and negative expression differences associated with gbM loss. However, expression is largely unaffected by gbM loss in *E. salsugineum*. Expression differences between species were within the variation of expression observed within *A. thaliana* accessions that displayed variation in gbM. Furthermore, experimentally induced loss of gbM did not consistently lead to differences in expression compared to wild type. To date, there is no convincing data to support a direct causal link between the presence/absence of gbM and the modulation of expression in flowering plants.

Gene body methylation (gbM) is characterized by the enrichment of CG methylation levels within coding regions (Tran *et al.* 2005; Zhang *et al.* 2006; Zilberman *et al.* 2007; Cokus *et al.* 2008; Lister *et al.* 2008; Takuno and Gaut 2012). Compared to unmethylated (UM) genes, gbM genes tend to be moderately and constitutively expressed (Zhang *et al.* 2006; Lister *et al.* 2008; Niederhuth *et al.* 2016). Many have speculated on the function of gbM, suggesting that its primary role is likely to be homeostatic (Zilberman 2017; Muyle and Gaut 2019). The discovery of species without gbM led to testing hypotheses for its function and evolutionary origin (Bewick *et al.* 2016; Bewick *et al.* 2017). No obvious and consistent consequences to gene expression were observed following the loss of gbM in *Eutrema salsugineum* (Bewick *et al.* 2016). However, a reanalysis of Bewick, *et al.* (2016) reported a small, but statistically significant reduction in gene expression associated with genes that have lost gbM in one of the two *E. salsugineum* accessions (Muyle and Gaut 2019). To say the least, the function of gbM remains enigmatic.

## Methods

### Datasets

Whole Genome Bisulfite Sequencing (WGBS) and RNA-seq data were downloaded from Gene Expression Omnibus (GEO) or Short Read Archive (SRA) for *Arabidopsis thaliana* and *Eutrema salsugineum*. For *A. thaliana* we selected six additional accessions to Col-0 that spanned the gamut of CG DNA methylation levels for gbM genes (∼30–60%) (Kawakatsu *et al.* 2016). WGBS and RNA-seq data from these additional accessions represent a single replicate per accession. However, comparison between wild type and *met1* epiRIL was performed in triplicate (Bewick *et al.* 2016). For *E. salsugineum* we selected the two available genotypes with available WGBS and RNA-seq (Bewick *et al.* 2016). GEO or SRA accession numbers and mapping statistics are located in Table S7.

### Identification of putative orthologs

Basic Local Alignment Search Tool (BLAST) (Altschul *et al.* 1990) was to identify putative orthologs between *A. thaliana* and *E. salsugineum*. We used identical BLAST parameters as Bewick *et al.* (2016) to recover the same set of putative one-to-one orthologs. Orthologs were then split into Group 1 (putative orthologous pairs that changed gbM status in *E. salsugineum*) and Group 2 (putative orthologous pairs that are UM in both *A. thaliana* and *E. salsugineum*) based on DNA methylation status in each species (see subsection **WGBS mapping and analysis of DNA methylation** for classification of gbM and UM). For Group 1, genes were required to be gbM across all *A. thaliana* accessions and UM for both *E. salsugineum* genotypes. Similarly, for Group 2 genes were required to be UM across all *A. thaliana* accessions and UM for both *E. salsugineum* genotypes. The number of genes within each group were lower than previously reported by Bewick *et al.* (2016) and Muyle and Gaut (2019) because of the DNA methylation requirements across accessions and genotypes, and the incorrect *A. thaliana-E. salsugineum* one-to-one orthology assignment of 1,301 genes by Muyle and Gaut (2019).

### WGBS mapping and analysis of DNA methylation

WGBS data were aligned to each species respective genome assembly using the methylpy pipeline (Schultz *et al.* 2015). In brief, reads were trimmed of sequencing adapters using Cutadapt v1.9 (Martin and Marcel 2011), and then mapped to both a converted forward strand (cytosines to thymines) and converted reverse strand (guanines to adenines) using bowtie v1.1.1 (Langmead *et al.* 2009). Reads that mapped to multiple locations, and clonal reads were removed. Additionally, PCR-duplicated reads were identified and removed using Picard v2.16.0 (https://github.com/broadinstitute/picard). Unmethylated lambda phage DNA or plastid genome was used as a control for sodium bisulfite conversion. Non-conversion rates are located in Table S7.

Weighted DNA methylation was calculated for CG sites by dividing the total number of aligned methylated reads by the total number of methylated plus unmethylated reads (Schultz *et al.* 2012). CG sequence context enrichment for each gene was determined through a binomial test followed by Benjamini-Hochberg false discovery rate following Takuno and Gaut (2012). A background mCG level was determined from all coding sequence, which was used as a threshold in determining significance with a False Discovery Rate (FDR) correction. Genes were classified as gene body methylated (gbM) if they had at least 3 reads mapping to 20 CG sites and a CG q-value ≤ 0.05. Genes were classified as unmethylated (UM) if they had reads mapping to at least 20 reads mapping to 20 CG sites and a CG, CHG, and CHH (H = A, C, or T) q-value > 0.05.

### RNA-seq mapping

Raw RNA-seq FASTQ reads were trimmed for adapters and preprocessed to remove low-quality reads using Trimmomatic v0.33 (arguments: default) (Bolger *et al.* 2014) prior to mapping to the species respective genome assembly. Reads were mapped using HISAT2 v2.1.0 (Pertea *et al.* 2016) supplied with a reference GTF (General Transfer Format) and splice-site and exon information (arguments: default). Following mapping, RNA-Seq alignments were assembled into potential transcripts using StringTie v1.3.3b (Pertea *et al.* 2016) (arguments: default).

### Analysis of gene expression

Differentially expressed genes between *met1* epiRIL and wild-type libraries were determined using edgeR v3.20.1 (Robinson and Oshlack 2010; Robinson *et al.* 2010) implemented in R v3.2.4 (https://www.r-project.org/). Mutant and wild-type libraries were collectively normalized using the Trimmed mean of M values (TMM) method (Robinson and Oshlack 2010). Genes were retained for DEG analysis if they possessed a count per million (CPM) ≥ 1 in at least ≥ 2 libraries. Significance was determined using the glmQLFTest function, which uses empirical Bayes quasi-likelihood F tests, and an FDR cutoff of 5%. Parameter settings were determined following best practices for DEG analysis as described by (Chen *et al.* 2016).

A bootstrap test with 1,000 replicates was used to determine significance of gene expression distributions for Group 1 between *A. thaliana* accessions and *E. salsugineum* genotypes, *E. salsugineum* genotypes, and for Group 1 and Group 2 between wild type and *met1* epiRIL. A Wilcoxon signed-rank test was also used to compare expression between pairs of genes in Group 1 (Table S8). Paired tests are appropriate when the data are not independent (*i.e.*, shared ancestry) and when the dependency results in a one-to-one match (*i.e.*, single-copy putative orthologous gene pairs). Both the statistical significance (*P* value) and the substantive difference (effect size) are important in the interpretation of studies (Sullivan and Feinn 2012). Hence, in addition to reporting statistical significance, we used Cohen’s *d* to assess effect size of difference in gene expression within Groups 1 and 2.

### Data Availability

All data are available at GEO or SRA and the accession numbers are listed in Table S7. Supplemental material available at FigShare: https://doi.org/10.25387/g3.8204582.

## Results and Discussion

To test for a function of gbM in modulating gene expression (Fragments Per Kilobase of transcript per Million mapped reads [FPKM]), Muyle and Gaut (2019) contrasted gene expression (log_10_[FPKM+1]) of two groups of genes. Group 1 genes consisted of putative orthologous pairs that changed gbM status in *E. salsugineum* (*i.e.*, gbM→UM) since its last common ancestor with *Arabidopsis thaliana*. Group 2 genes consisted of putative orthologous pairs that are UM in both *A. thaliana* and *E. salsugineum*. This comparison (i) does not directly test the function of gbM in modulating gene expression and (ii) introduces unlikely assumptions regarding gene expression. First, Muyle and Gaut’s (2019) model is designed to test for an effect of Group on gene expression, rather than a change in gene expression following the loss of gbM. Second, Group 2 is an inappropriate control to test for the function of gbM in modulating gene expression. Using Group 2 as a control incorrectly assumes that gene expression levels of UM genes have remained relatively constant over millions of years. This assumption is contrary to the proposed homeostatic function of gbM, where under this model, it is predicted that UM genes have more variable gene expression compared to orthologous gbM genes (Zilberman 2017).

**Figure 1:**
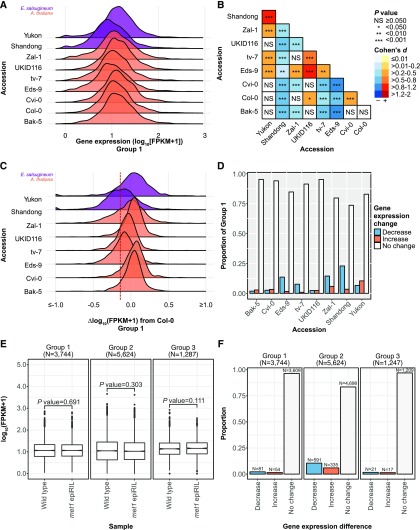
No direct and consistent consequences to gene expression following the loss of gbM. (A) Ridge plot of gene expression for the same 1,328 genes across accessions and genotypes. All genes have lost gbM in *E. salsugineum* Shandong and Yukon, whereas all genes maintain gbM in *A. thaliana* accessions (Group 1). (B) Pair-wise comparison of gene expression for Group 1 between species (*E. salsugineum-A. thaliana*) and within species (*E. salsugineum-E. salsugineum* and *A. thaliana-A. thaliana*). Significance is represented by asterisks. Color of cell indicates direction and effect size of difference relative to the x-axis. The effect size is represented as Cohen’s *d*. (C) Ridge plot of gene expression differences for each genotype and accession relative to *A. thaliana* Col-0. Gene expression differences ≤-1.0 and ≥1.0 log_10_(FPKM+1) were collapsed to -1.0 and 1.0, respectively. Dashed red line indicates the average difference in gene expression reported by Muyle and Gaut (2019) for Group 1 genes between *E. salsugineum* and *A. thaliana* Col-0 (-0.14 log_10_[FPKM+1]). (D) Bar plot of discrete differences in genes expression for each accession and genotype relative to *A. thaliana* Col-0. (E) Distribution of gene expression for Group 1, Group 2, and Group 3 genes between wild type and *met1* epiRIL. Gene expression was averaged per gene across three wild type and *met1* epiRIL RNA-seq libraries, respectively. Boxplot elements: center line, median; upper and lower “hinges”, first and third quartiles (the 25^th^ and 75^th^ percentiles), respectively; whiskers, 1.5× interquartile range; large points, outliers. (F) Bar plot of discrete differences in genes expression for Group 1, Group 2, and Group 3 genes between wild type and *met1* epiRIL.

If gbM modulates gene expression, a consistent effect on gene expression would be expected upon loss of gbM. However, only one of the two *E. salsugineum* genotypes tested in the Muyle and Gaut (2019) study supported the conclusion that gene expression is reduced following loss of gbM. This raises the possibility that the gene expression variation observed by Muyle and Gaut (2019) is within the expected range for expression variation between any group of orthologous genes that have been diverging for millions of years. To test this hypothesis further, we used data from the 1,001 *A. thaliana* epi/genomes project (1001 Genomes Consortium 2016; Kawakatsu *et al.* 2016). Expression levels of gbM genes from seven *A. thaliana* accessions and their putative orthologs from two *E. salsugineum* genotypes that have lost gbM (N = 1,328) show substantial overlap (Figure 1A and Table S1). Mean gene expression and standard error of the mean (SEM) of *E. salsugineum* Yukon fall within the range of *A. thaliana* accessions investigated (Table S2). However, mean gene expression and SEM of *E. salsugineum* Shandong is the lowest of genotypes and accessions investigated (Table S2). Gene expression consequences following the loss of gbM are inconsistent across *E. salsugineum* genotypes when compared to *A. thaliana* accessions (Figure 1B). Gene expression is higher in *E. salsugineum* Yukon compared to *A. thaliana* accessions, with several nonsignificant exceptions, whereas gene expression is lower in *E. salsugineum* Shandong compared to *A. thaliana* accessions. Differences in gene expression between *A. thaliana* accessions for gbM genes is also observed, but several comparisons are nonsignificant (Figure 1B). The effect size of gene expression differences between accessions, as measured using Cohen’s *d*, ranges from small to large for between and within species comparisons. These data fail to support that gene expression is reduced following the loss of gbM and instead suggest that the observed differences between *E. salsugineum* and *A. thaliana* are within an expected distribution of natural variation in gene expression.

Muyle and Gaut (2019) report an average 0.14 log_10_(FPKM+1) decrease following the loss of gbM in *E. salsugineum*. Similarly, to Muyle and Gaut (2019), we compared Group 1 gene expression between *E. salsugineum* genotypes and *A. thaliana* Bak-5, Cvi-0, Eds-9, tv-7, UKID116, and Zal-1 to *A. thaliana* Col-0 using the exact same methods. Compared to *A. thaliana* Col-0, both increases and decreases to gene expression are observed for genes that lost gbM in either *E. salsugineum* and/or possess gbM in *A. thaliana* accessions (Figure 1C). Although *E. salsugineum* Shandong shows an on average negative difference in gene expression identical to that reported by Muyle and Gaut (2019) (-0.138 log_10_[FPKM+1]), *E. salsugineum* Yukon shows an average positive difference in gene expression (+0.017 log_10_[FPKM+1]). However, in *E. salsugineum* Shandong and Yukon, 53.24% and 82.53% of genes that have lost gbM show an increase in gene expression greater than the 0.14 log_10_(FPKM+1) reduction reported by Muyle and Gaut (2019).

Furthermore, requiring a ≥1 (increase) or ≤-1 (decrease) log_2_ difference in gene expression, more genes that have lost gbM have higher rather than lower expression in *E. salsugineum* Yukon compared to *A. thaliana* Col-0 gbM genes (Figure 1D). However, the majority of genes that have lost gbM in *E. salsugineum* and gbM genes in *A. thaliana* accessions Bak-5, Cvi-0, Eds-9, tv-7, UKID116, and Zal-1 are not differentially expressed given this threshold compared to *A. thaliana* Col-0 gbM genes (Figure 1D). Differences in sequencing coverage between *A. thaliana* Col-0 and *E. salsugineum* may interfere with gene expression comparisons, particularly if there are heterogeneous transcript distributions between samples (Bullard *et al.* 2010; Hansen *et al.* 2010). However, equalizing sequencing coverage of genes between *A. thaliana* Col-0 and *E. salsugineum* (*e.g.*, Shandong) has no effect on gene expression levels, suggesting transcript heterogeneity is trivial and is not introducing biases to these comparisons (Fig. S1). Furthermore, the on average negative difference in gene expression between *A. thaliana* Col-0 and *E. salsugineum* Shandong for Group 1 is similar to the difference between *E. salsugineum* Shandong and Yukon for all genes (-0.170 log_10_[FPKM+1]) (Fig. S2), suggesting an outgroup effect influencing differences in gene expression. Collectively these results demonstrate gene expression differences following the loss of gbM in *E. salsugineum* are within the range of differences between gbM genes of *A. thaliana* accessions. These results further support an inconsistent consequence to differences in gene expression following the loss of gbM.

Gene expression comparison between species could be confounded by inherent experimental and biological biases. Hence, to test for direct differences to gene expression following the loss of gbM, while mitigating biases, we took advantage of the availability of eighth-generation *met1* epigenetic Recombinant Inbred Lines (epiRILs) (Reinders *et al.* 2009). The *met1* epiRILs contain mosaic methylomes, including genomic regions with normal DNA methylation from the wild-type parent and gbM-free regions from the *met1* parent. No difference in gene expression is observed for Group 1 genes between the *met1* epiRIL and wild type (Figure 1E and Table S3). Using the expression difference threshold as above, we identified 31/3,744 (0.83%) and 22/3,744 (0.59%) Group 1 genes experiencing a positive and negative difference in gene expression, respectively. To account for the slight possibility of transcript heterogeneity between *met1* epiRIL and wild type, we collectively normalized gene expression across samples and replicates using the Trimmed mean of M values (TMM) method prior to the identification of Differentially Expressed Genes (DEGs) in edgeR v3.20.1 (Robinson and Oshlack 2010; Robinson *et al.* 2010). This more formal approach to compare gene expression differences recovered a total of 1,061 DEGs with 135 (12.72%) belonging to Group 1 and 926 (87.28%) belonging to Group 2 (Table S4). Gene expression differences are not independent of Group (χ^2^ = 6.255e-06, d.f. = 1, *P* value = 1.788e-82) with fewer gene expression differences following the loss of gbM being observed than expected by chance (N_Expected_ = 424) (Table S5). Among the 135 DEGs in Group 1, 81 have decreased and 54 have increased gene expression levels in the *met1* epiRIL relative to wild type (Figure 1F and Table S6). Similarly, among the 926 DEGs in Group 2 genes, 591 show decreased and 335 show increased expression in the *met1* epiRIL relative to wild type (Figure 1F and Table S6). Hence, there are similar negative and positive gene expression differences between Groups 1 and 2 (∼3:2). We also compared gene expression of genes that are gbM in both *met1* epiRIL and wild type (Group 3). Group 3 genes are similar to Group 1 genes with respect to gene expression level and the proportion of DEGs (Figure 1E and F). Overall, these results show the loss of gbM does not lead to direct and consistent differences to gene expression. Instead, gene expression differences that are observed for Group 1 genes are likely indirect effects associated with the gain and recovery of MET1.

The association of gbM to gene expression is often interpreted as it is possessing a functional role, however, there is currently no direct evidence to support this role. Evolutionary loss of gbM without clear consequences to gene expression suggests a lack of a role in transcriptional regulation, but this too is an indirect test which requires further support. In contrast, experimentally induced loss of gbM in the *met1* epiRIL did not lead to significant differences in gene expression and the few genes with significant differences to gene expression were both negative and positive. These data suggest that the function of gbM is not likely modulating or buffering gene expression. Zilberman (2017) proposes that the primary effect of gbM is likely to be homeostatic, possibly by preventing aberrant transcription within genes or restricting access to histone H2A.Z. However, the pattern of H2A.Z in *E. salsugineum* Shandong and *met1* epiRIL remains comparable to that in wild type (Bewick *et al.*, 2016). If a functional role of gbM exists, it has yet to be convincingly determined. Continued discussions and research on this curious feature of flowering plant genomes will help unravel this mystery.
